# LAIR-1 suppresses cell growth of ovarian cancer cell via the PI3K-AKT-mTOR pathway

**DOI:** 10.18632/aging.103589

**Published:** 2020-08-31

**Authors:** Yan Liu, Li Ma, Fugen Shangguan, Xuena Zhao, Wenjie Wang, Zhiyue Gao, Huimin Zhou, Guiwu Qu, Yumei Huang, Jing An, Jiangnan Xue, Shude Yang, Qizhi Cao

**Affiliations:** 1 Department of Immunology, School of Basic Medical Sciences, Binzhou Medical University, Yantai 264003, Shandong, P.R. China; 2 Key Laboratory of Diagnosis and Treatment of Severe Hepato-Pancreatic Diseases of Zhejiang Province, The First Affiliated Hospital of Wenzhou Medical University, Wenzhou 325000, P.R. China; 3 Binzhou Medical University, Yantai 264003, Shandong, P.R. China; 4 Anti-aging Research Institution, Binzhou Medical University, Yantai 264003, Shandong, P.R.China; 5 Department of Stomatology, Affiliated Hospital of Binzhou Medical College, Binzhou 256603, Shandong, P.R. China; 6 Division of Infectious Diseases and Global Health, School of Medicine, University of California at San Diego, La Jolla, CA 92037, USA; 7 School of Agriculture, Ludong University, Yantai 264025, Shandong, P.R.China; 8 Equal contribution

**Keywords:** LAIR-1, ovarian cancer, PI3K-AKT-mTOR pathway, eEF1A2, cell proliferation

## Abstract

Recently, over-expression of LAIR-1 has been found in some solid cancers, including ovarian cancer. The role of LAIR-1 in cancer progression needs further investigation. In this study, we identified the LAIR-1 cDNA sequence of the ovarian cancer cells HO8910. Using SKOV3 cells, we confirmed the finding from our previous study that LAIR-1 could suppress in vitro cell proliferation and cell migration. We also found LAIR-1 overexpression can induce apoptosis of SKOV3 cells. We revealed LAIR-1 suppressed cell growth by inhibiting the PI3K-AKT-mTOR axis. Moreover, the LAIR-1 antitumor activity and its mechanism were also identified in vivo. We used Co-IP assay and mass spectrometry to identify potential LAIR-1-binding proteins in LAIR-1 overexpressing SKOV3 cells. MS analysis identified 167 potentially interacting proteins. GO analyses indicated a possible involvement of LAIR-1 in mRNA processing through its interaction with some eukaryotic translation initiation factors (eIF4E1B, eIF2S3, eIF3D, eIF4G2, eIF5B) and eukaryotic translation elongation factors (eEF1A2 and eEF1B2). Our findings suggest that LAIR-1 may suppress the growth of ovarian cancer cells by serving as a modulator that suppresses PI3K-AKT-mTOR directly or regulating protein synthesis at the translational level. Our results indicate that a LAIR-1-based strategy may prevent or suppress the progression of ovarian cancer.

## INTRODUCTION

Ovarian cancer is the fifth greatest cause of cancer-related death in women. Most patients present at an advanced clinical stage when curative therapy is no longer possible [1]. Therefore, improvement in patient outcomes will require identification of the molecular mechanisms controlling cancer progression and discovery of new biomarkers and therapeutic opportunities.

One potential biomarker is leukocyte-associated immunoglobulin-like receptor 1 (LAIR-1), an immune inhibitory receptor that plays an inhibitory role in the maturation, differentiation and activation of immune cells, including NK cells, T cells, B cells and monocytes [2–5]. In our previous study, we found that LAIR-1 is expressed in ovarian cancer tissues and in several ovarian cancer cell lines, including COC1 and HO8910 cells. The expression of LAIR-1 in ovarian cancer tissues was significantly correlated with tumor grade, and was involved in the proliferation and invasion of HO8910 cells [6]. In recent years, over expression of LAIR-1 has been reported in cervical cancer, oral squamous cell carcinoma and hepatocellular carcinoma. In those studies, the LAIR-1 expression level was significantly associated with tumor pathological differentiation, in agreement with our previous results for ovarian cancer [7–9]. Taken together, these findings suggest that LAIR-1 may have a special role in tumors of non-hematopoietic lineage. However, the precise mechanism by which LAIR-1 influences the biological functions of carcinoma cells is still unclear.

In the present study, we identified the LAIR-1 cDNA sequence of the ovarian cancer cells HO8910. Using SKOV3, which is another ovarian cancer cell line expressing lower level of LAIR-1, we confirmed in vitro the effects of LAIR-1 on cell proliferation, colony formation and cell invasion. We investigated the effect of LAIR-1 overexpression on the apoptosis of SKOV3 cells as well. Furthermore, we investigated the molecular mechanisms in ovarian cancer cells, and studied the LAIR-1 antitumor activity and its mechanism in vivo. We have demonstrated for the first time that LAIR-1 is involved in the regulation of the PI3K-AKT-mTOR pathway that regulates the cell proliferation, apoptosis and metabolism of normal cells and is implicated in many cancers, including ovarian cancer [10–14]. We also used Co-Immunoprecipitation (Co-IP) assay and mass spectrometry to identify potential LAIR-1-binding proteins in LAIR-1-overexpressing SKOV3 cells. Further analyses of interacting proteins should aid in elucidating the biological functions of LAIR-1 in the progression of ovarian cancer.

## RESULTS

### DNA cloning of the LAIR-1 mRNA transcript from HO8910 cells

Previous studies on human NK cells have reported that the expression of two distinct forms of LAIR-1 with molecular weights of ~40 KDa and ~34 KDa; by contrast, human T cell tumor Jurkat cells express the ~34 KDa form exclusively. The cDNAs encoding the ~40 KDa and ~34 KDa LAIR-1 are designated as LAIR-1a and LAIR-1b, respectively. Our previous study also showed expression of both forms of LAIR-1 in HO8910 cells, but greater expression of the longer form. The LAIR-1a sequence data from one NK clone is available from EMBL/GenBank/DDBJ under accession numbers AF013249 and NM_002287.4. This 864-bp open reading frame encodes a type I membrane protein with a 21-aa leader sequence, a 142-aa extracellular domain, a 23-aa transmembrane domain, and a 101-aa cytoplasmic region containing two ITIM sequences VTYAQ and ITYAAY [15, 16]. At present, six LAIR-1 transcript variants (a, b, c, e, f, and g) have been reported in the NCBI database (https://www.ncbi.nlm.nih.gov/gene/3903), and the sequence of NM_002287.4 has been updated into a current version NM_002287.6.

**Figure 1:**
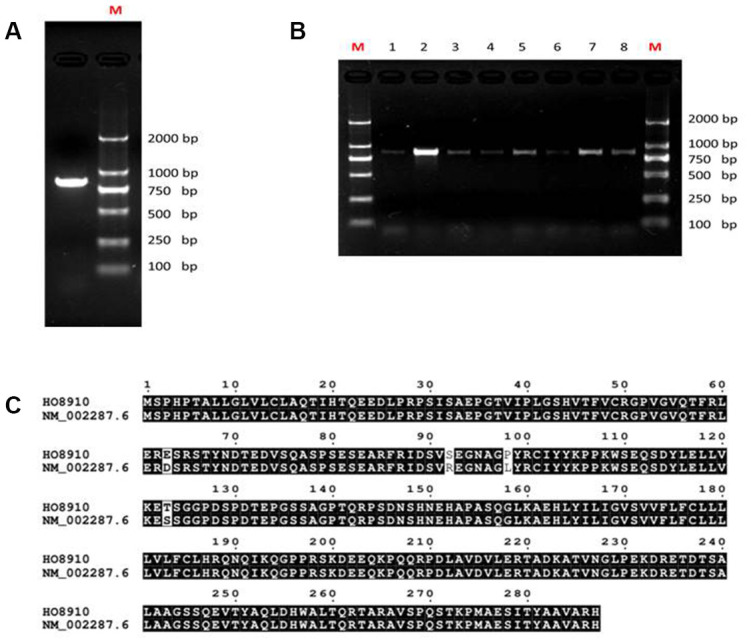
**Analysis of LAIR-1 cDNA sequence in HO8910 ovarian cancer cells.** (**A**) LAIR-1 PCR product analysis of HO8910 cells on 1% electrophoresis gel. (**B**) Colony PCR analysis was used to identify the recombinant positive colonies, the positive colonies were purified and subsequently sequenced. (**C**) The predicted amino acid sequence alignment between LAIR-1 in HO8910 cells and NM_002287.6.

The sequence of the LAIR-1 gene in HO8910 cells was studied by isolating total RNA from the cells and then amplifying the cDNA encoding LAIR-1 using primers designed according to the longest LAIR-1 transcript variant a (NM_002287.6). As shown in Figure 1A, only one cDNA band was obtained. The PCR products were TA cloned and transfected into *E. coli* DH5α cells. Recombinant clones were identified using colony PCR (Figure 1B) and were completely sequenced in two directions. We obtained one 866-bp DNA sequence containing an 864-bp open reading frame, this nucleotide sequence was aligned to that of LAIR-1a (AF013249; NM_002287.4). The alignment result showed 100% identity between the LAIR-1 cDNA sequence of HO8910 and that of LAIR-1a [15]. In our previous study, we cloned and sequenced the LAIR-1 cDNA of COC1, another ovarian cancer cell line expressing high level LAIR-1. The cDNA sequence of COC1 also shared 100% identity with that of HO8910 (Date not shown). The predicted amino acid sequence of LAIR-1 in HO8910 shared 98.61% identity with that of NM_002287.6 (Figure 1C).

### LAIR-1 inhibits SKOV3 cell growth and migration and induces apoptosis in vitro

**Figure 2:**
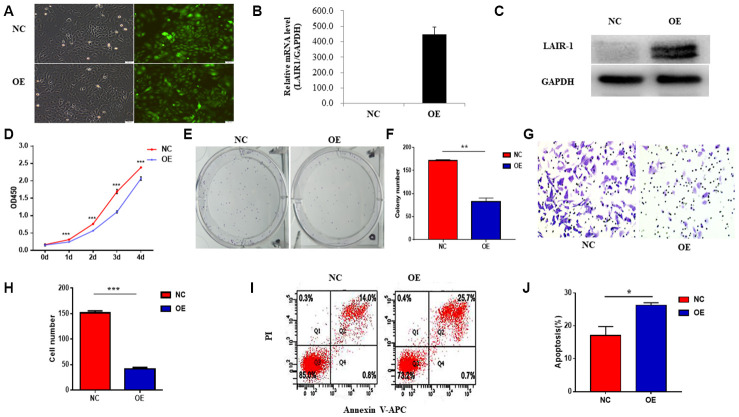
**LAIR-1 inhibits SKOV3 cells growth and promotes cell apoptosis in vitro.** (**A**) SKOV3 cells were transfected with control or LAIR-1 overexpressed lentivirus, the efficiency of LAIR-1 overexpression was examined by fluorescence microscope. (**B**, **C**) qRT-PCR and western blot were also used to further confirm the efficiency of LAIR-1 overexpression in SKOV3 cells. GAPDH was used as a loading control. (**D**–**F**) The cell growth was measured by cell proliferation assay and colony formation assay in control and LAIR-1 overexpressed SKOV3 cells. (**G**, **H**) Cell migration ability was assessed by transwell migration assay in control and LAIR-1 overexpressed SKOV3 cells. (**I**, **J**) Flow cytometry analysis of cell apoptosis in control and LAIR-1 overexpressed SKOV3 cells. Cells were collected and stained with Annexin V-fluorescein isothiocyanate (FITC) and PI. Data are presented as mean ± SD.

In our previous study, we showed that knockdown of LAIR-1 promotes HO8910 cell proliferation, colony formation and invasive ability. We confirmed the effect of LAIR-1 on the malignant phenotypes of other ovarian cancer cells by stably overexpressing LAIR-1 in SKOV3, an ovarian cell line with lower level LAIR-1 expression, using a LAIR-1-lentivirus based on the cDNA sequence of LAIR-1 from HO8910 and COC1. The efficiency of transfection with LAIR-1-lentivirus in SKOV3 cells was shown in Figure 2A. The overexpression of LAIR-1 was confirmed by qRT–PCR (Figure 2B) and western blotting (Figure 2C).

Cell proliferation assays using a CCK-8 kit revealed that overexpression of LAIR-1 significantly inhibited SKOV3 cell proliferation (Figure 2D). We also performed a colony formation assay to verify the anti-proliferative activity of LAIR-1. As shown in Figure 2E, 2F, LAIR-1 overexpression significantly reduced the clonogenic ability of SKOV3 cells.

The ability to invade surrounding tissues and metastasize to distant organs is an important hallmark of ovarian cancer cells. Therefore, we next examined whether LAIR-1 could affect in vitro the migratory properties of SKOV3 cells. Cell migration assays showed that LAIR-1 suppressed SKOV3 cell migration (Figure 2G–2H). Given the inhibitory effect of LAIR-1 on SKOV3 cell proliferation and migration, we also analyzed the impact of LAIR-1 on cell apoptosis. As shown in Figure 2I–2J, LAIR-1 promoted apoptosis in SKOV3. These data, combined with our previous study, indicated that LAIR-1 have an anti-tumorigenic role in ovarian cells.

### LAIR-1 suppresses ovarian cancer cell by regulating the PI3K-AKT-mTOR axis

The PI3K-AKT-mTOR pathway is one of the most frequently mutated or altered pathways in ovarian cancers and plays an important role in tumorigenesis, proliferation, and cancer progression. AKT and mTOR, which are the major components of this signaling pathway, are both elevated in ovarian cancers [12–14, 17, 18]. We therefore hypothesized that LAIR-1 might affect the cell growth of SKOV3 and HO8910 cells by regulating this pathway.

**Figure 3:**
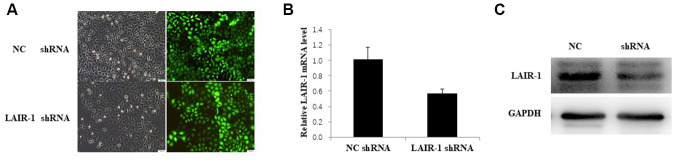
**LAIR-1 knockdown HO8910 cells were constructed by transfecting with LAIR-1-RNAi-lentivirus.** (**A**) HO8910 cells were transfected with control and LAIR-1shRNA lentivirus, the efficiency of LAIR-1 knockdown was examined by fluorescence microscope. (**B**, **C**) qRT-PCR and western blot were also used to further confirm the efficiency of LAIR-1 knockdown in HO8910 cells.

The PI3K-AKT-mTOR signal pathway was investigated in HO8910 cells by shRNA knockdown of LAIR-1 via transfection with LAIR-1-RNAi-lentivirus. The efficiency of transfection with LAIR-1-RNAi-lentivirus in HO8910 cells is shown in Figure 3A. The LAIR-1 knockdown efficiency was confirmed by qRT–PCR (Figure 3B) and western blotting (Figure 3C).

**Figure 4:**
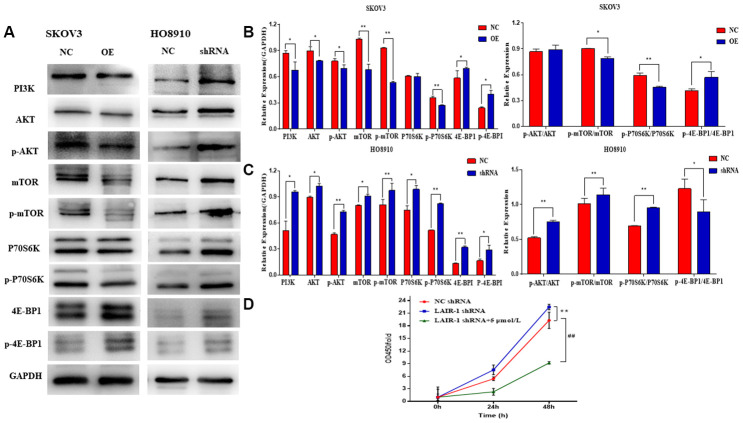
**LAIR-1 suppresses ovarian cancer cell growth by regulating PI3K-AKT-mTOR signal axis.** (**A**) Western blot analyses of PI3K, AKT, p-AKT, mTOR, p-mTOR, P70S6K, p-P70S6K, 4E-BP1 and p-4E-BP1 levels in control and LAIR-1 overexpression SKOV3 cells, control and LAIR-1 knockdown (shRNA) HO8910 cells. (**B**, **C**) These proteins were quantified according to the gray value of each band. And the data expressed in right graphs represent the mean ± SD in both SKOV3 and HO8910 cells. (**D**) The control and LAIR-1 knockdown HO8910 cells were treated with or without PI3K inhibitor LY294002, and then the cell viabilities was detected by CCK-8 assay. Cell growth curves were plotted according to the OD values. Data are presented as the mean ± SD (**p < 0.01, ^##^p < 0.01).

Western blot analysis showed that PI3K, AKT, and mTOR expression and the phosphorylation of both AKT (p-AKT) and mTOR (p-mTOR) were significantly decreased in LAIR-1 overexpressing SKOV3 cells, whereas they were increased after shRNA knockdown of LAIR-1 in HO8910 cells. The same results were obtained for the phosphorylation ribosomal protein S6 kinase (P70S6K), one effector of mTOR. We also detected the expression of eIF4E-binding protein 1 (4E-BP1), another target of the AKT-mTOR pathway. In parallel with the expression of LAIR-1 in SKOV3 cells, the expression of 4E-BP1, the phosphorylation of 4E-BP1 (p-4E-BP1), and the ratio of p-4E-BP1/4E-BP1 increased significantly in LAIR-1 overexpressing SKOV3 cells compared with control cells. By contrast, the p-4E-BP1/4E-BP1 ratio decreased significantly after shRNA knockdown of LAIR-1 in HO8910 cells (Figure 4A–4C). These data demonstrated that LAIR-1 suppresses cell growth via the PI3K-AKT pathway in vitro. We further explored whether PI3K-AKT inhibitor can reverse the effects from LAIR-1 in ovarian cancer cells by evaluating the growth of HO8910 cells treated with or without a PI3K inhibitor LY294002. Our data indicated LY294002 efficiently inhibited the cell growth of HO8910 cells induced by shRNA knockdown of LAIR-1 (Figure 4D). These results indicate that LAIR-1 affects ovarian cancer cell growth via the PI3K-AKT pathway.

### LAIR-1 inhibits SKOV3 cell growth in vivo

**Figure 5:**
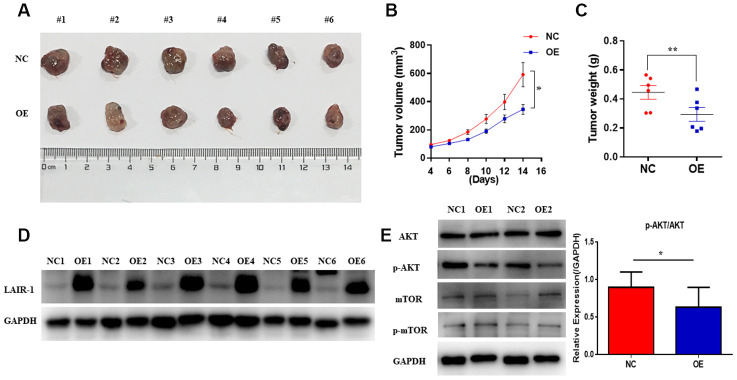
**LAIR-1 inhibits ovarian cancer cell growth in vivo.** Nude BALB/c mice were subcutaneously injected with control (NC) and LAIR-1 overexpression SKOV3 cells (OE). (**A**) The tumor tissues obtained from mice were imaged using a digital camera (**B**). Tumor volume in each mouse was monitored every two days. Data are presented as mean ± SE (n = 6). (**C**) The tumors tissues were weighed by analytical balance. Data are presented as mean ± SE (n = 6). (**D**) Western blot analyses of LAIR-1 expression in tumor tissues derived from control and LAIR-1 overexpression SKOV3 cells. GAPDH was used as a loading control. (**E**) Western blot analyses of p-AKT, AKT, p-mTOR, mTOR pathways in tumor tissues derived from control and LAIR-1 overexpression SKOV3 cells. GAPDH was used as a loading control.

A mouse SKOV3 xenograft model of ovarian cancer was employed to further investigate the in vivo anti-tumorigenic potential of LAIR-1. SKOV3 cells infected with LAIR-1-lentivirus (OE) or control-lentivirus (NC) were injected subcutaneously into the left flank of 5-week-old mice (n = 6/each group). After 14 days, the mice were sacrificed, and the tumors were dissected and weighed. As shown in Figure 5A and 5B, overexpression of LAIR-1 significantly inhibited in vivo SKOV3 cell growth and reduced the tumor weight (Figure 5C). We next analyzed AKT-mTOR pathway in tumor samples. Consistently, LAIR-1 overexpression could significantly down-regulate the p-AKT/AKT ratio (Figure 5E). To understand the extent to which the tumor suppressive function of LAIR-1 in vivo is related to inhibition of cell proliferation or apoptosis, we conducted further western blotting studies to determine the expression levels of key markers associated with cell proliferation (ki-67 and PCNA) and apoptosis (Bcl-2 and Bax). Bcl-2 markedly decreased, leading to a dramatic increase in the Bax/Bcl-2 ratio in LAIR-1 overexpression tumor samples (Supplementary Figure 1), while the expression levels of ki-67 and PCNA remained unchanged. Taken together, these results suggest that LAIR-1 overexpression inhibits SKOV3 cell growth in vivo mainly due to apoptosis via the regulation of the PI3K-AKT pathway and coordinated Bcl-2 family proteins.

### Identification and function annotation of LAIR-1 binding proteins

**Figure 6:**
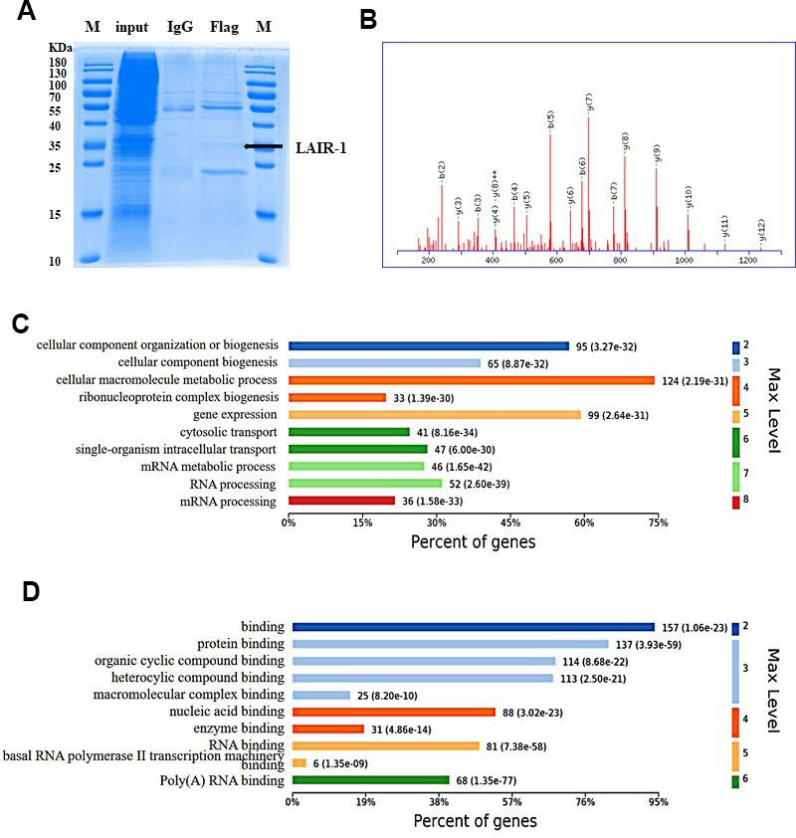
**Identification and GO enrichment analysis of LAIR-1 binding proteins.** The potential LAIR-1 binding protein was identified using Co-IP assay and mass spectrometry. (**A**) Coomassie brilliant blue staining of polyacrylamide gels loaded with the Co-IP samples. (**B**) Mass spectrum of one unique peptide THINIVVIGHVDSGK found in sp|Q05639|EF1A2_HUMAN, the most abundant LAIR-binding protein. (**C**, **D**) Top 10 significantly enriched GO terms in biological process and molecular function are displayed, respectively. Terms of same category are ordered by annotated level. Max Level: maximal annotated level of the corresponding term in the GO graph (tree). Information on percentage of proteins involved in the term is shown on the x-axis.

Above results showed that LAIR-1 overexpression suppressed the cell proliferation and migration of SKOV3 cells, and was involved in the regulation of the PI3K-AKT-mTOR pathway. We examined the underlying mechanism using Co-IP assay and mass spectrometry to identify potential LAIR-1-binding proteins. Coomassie brilliant blue staining of polyacrylamide gels loaded with the Co-IP samples was shown in Figure 6A. GO pathway analysis was conducted on the http://www.omicsbean.cn to explore the potential biological functions of LAIR-1.

A total of 167 proteins were identified as potential LAIR-1-binding proteins by LC-ESI-MS/MS. EF1A2 was the top protein, according to its abundance in the MS data. Mass spectrum of one unique peptide THINIVVIGHVDSGK found in sp|Q05639|EF1A2_HUMAN was shown in Figure 6B. The GO analysis of the biological processes and molecular functions revealed the main enriched GO biological processes to be mRNA processing, RNA processing, and mRNA metabolic processing (Figure 6C). The main enriched GO molecular functions included poly(A) RNA binding, RNA binding, and basal RNA polymerase II transcription machinery binding (Figure 6D). This information suggested that LAIR-1 might be involved in mRNA regulation in RNA binding and processing.

## DISCUSSION

Up-regulation of LAIR-1 expression in many solid cancers, including cervical and ovarian cancers, has been reported in several studies recently [6–9]. The effects and molecular mechanism of LAIR-1 in the progression of cancer needs further investigation.

In the present study, we cloned and identified the cDNA sequence of LAIR-1 from the HO8910 ovarian cancer cells. Our results confirmed that this sequence was 100% identical to that of LAIR-1 from COC1 (another ovarian cell line) and from one NK cell clone [15]. We showed that LAIR-1 could suppress ovarian cancer cell growth both in vitro and in vivo via the PI3K-Akt-mTOR pathway. The PI3K-Akt-mTOR signaling pathway plays an important role in maintaining normal cell functions. PI3K, Akt and mTOR are the three major nodes in this pathway, and their deregulation plays a crucial role in the pathogenesis of many human cancers including ovarian cancer [12–14, 17, 18]. In both HO8910 and SKOV3 cells, western blot analyses of PI3K, p-AKT, AKT, p-mTOR and mTOR indicated an association between the expression of LAIR-1 and the regulation of the PI3K-AKT-mTOR axis. We confirmed this result by using a PI3K inhibitor, LY294002, which can efficiently reverse the effects of LAIR-1 expression in ovarian cancer cells. In addition, the LAIR-1 overexpression SKOV3 cells showed a significant decrease in the expression of P70S6K, one of the downstream effectors of the PI3K/AKT signal transduction pathway. P70S6K phosphorylates the S6 protein of the 40S ribosomal subunit and plays a positive role in protein synthesis and cell growth [12, 19]. Another effector of this signal pathway, 4E-BP-1, has tumor suppressor activity by blocking mRNA translation, thereby inhibiting protein synthesis and cell proliferation. In its non-phosphorylated active form, 4E-BP1 inhibits cap-dependent translation initiation by binding eukaryotic translation initiation factor 4E (eIF4E) and prohibiting the formation of the 48S pre-initiation complex, thereby restricting translational activity [20]. We observed that LAIR-1 could promote the non-phosphorylated 4E-BP1 expression in the SKOV3 cells. Moreover, LAIR-1 overexpression promoted SKOV3 cell apoptosis both in vitro and in vivo. A significant Bcl-2 down expression and a consequent increase in the Bax/Bcl-2 ratio in LAIR-1 overexpression tumor samples suggests that apoptosis via the regulation of the PI3K-AKT pathway and Bcl-2 family proteins may play an important role in the tumor suppressive function of LAIR-1 in vivo.

In order to understand more detail on the mechanism associated with LAIR-1, we further identified 167 LAIR-1 binding proteins by using co-IP assays and mass spectrometry. The most abundant LAIR-1 binding protein was eEF1A2, a protein that is overexpressed in 30% of ovarian tumors. The eEF1A2 protein is a translation elongation factor that delivers aminoacyl-transfer RNAs to the ribosome during translation. Recent research has identified eEF1A2 as a pro-oncogenic protein, can enhance cell invasion and migration in an Akt- and PI3K-dependent manner [21, 22], and can interact with p-Akt to regulate p-Akt levels [23]. Therefore, we speculate that an interaction between LAIR-1 and eEF1A may possibly suppress the AKT-mTOR pathway by direct regulation of the p-AKT level, but this needs further investigation.

The results of the GO enrichment analysis showed that mRNA processing is the main enriched biological process and that poly (A) RNA binding and RNA binding are the main enriched molecular functions. The LAIR-1 binding protein includes the eukaryotic translation initiation factors (eIF4E1B, eIF2S3, eIF3D, eIF4G2, and eIF5B) and the eukaryotic translation elongation factors eEF1A2 and eEF1B2. These findings indicate that LAIR-1 may regulate protein synthesis at the translational level through an interaction with these eukaryotic translation initiation or elongation factors. The translational process of protein synthesis is viewed as providing important clues for understanding oncogenesis. The interaction between LAIR-1 and these translation factors is also needed to understand in detail in the future.

In summary, we confirm the antitumor activity of LAIR-1 in ovarian cancer cells both in vitro and in vivo, which is associated with the suppression of the PI3K-AKT-mTOR pathway. The involved mechanisms may include LAIR-1 regulation of protein synthesis at the translational level or its action as a modulator that suppresses the PI3K-AKT-mTOR pathway directly. Ovarian cancer is a lethal gynecological cancer and The Cancer Genome Atlas (TCGA) data have revealed a hyperactivation of the PI3K-AKT-mTOR pathway in nearly 60% of patients with ovarian cancer. These aberrations provide strong support for approaches aimed at identifying potential therapeutic targets in the PI3K–AKT–mTOR signaling pathway [12, 24]. Our findings enhance the current understanding of the molecular mechanisms involved in LAIR-1 associated diseases. We previously mentioned that LAIR-1 is overexpressed in some solid cancers but not in normal tissues [6–9]. The differential expression pattern of LAIR-1 in tumors versus normal tissues does not contradict with our present results showing that overexpression of LAIR-1 suppresses cell growth and induces apoptosis in ovarian cancer. The LAIR-1 expression level is significantly associated with tumor pathological differentiation, which suggests LAIR-1 may be a poor-differentiation biomarker for this cancer and may play a role in control tumor differentiation [6]. Further investigation of the mechanism of LAIR-1in the regulation of tumor differentiation and progression will add another dimension to our understanding of its potential role in cancer.

## MATERIALS AND METHODS

### Cell lines and culture condition

Human ovarian cancer SKOV3 and HO8910 cell lines were purchased from the Shanghai Genechem Co., LTD (Shanghai, China). Both cell lines were maintained in the RPMI 1640 medium (Hyclone; GE Healthcare Life Sciences, Logan, UT, USA) supplemented with 10% fetal bovine serum (Gibco; Thermo Fisher Scientific, Inc., Waltham, MA, USA) and antibiotics (100 U/mL penicillin and 100μg/mL streptomycin) in a humidified incubator with 5% CO_2_ at 37°C.

### Reagents and antibodies

Anti-LAIR1 antibody [lc12] (ab14826) was purchased from Abcam (Cambridge, MA). All of the following primary antibodies were purchased from Cell Signaling Technology (USA): AKT (no. 9272S); p-AKT (no. 4060S); mTOR (no. 2983S); p-mTOR (no. 5536S); P70S6K (no. 2708S); p-P70S6K (no. 9234S); 4E-BPI (no. 9452S); p-4E-BPI (no. 9455S). Anti-Bcl-2 (no. 12789-1-AP), Bax (no. 50599-2-lg) and GAPDH (no. 10494-1-AP) antibodies were purchased from Proteintech (Wuhan, China). Anti-Ki67 (no. A2094) and β-Actin (no. AC026) were purchased from Abclonal (Wuhan, China). Horseradish peroxidase (HRP)-conjugated goat anti-mouse antibody and HRP-conjugated goat anti-rabbit antibody were purchased from Zhongshan Jinqiao Bio-technology Limited Company (Beijing, China). The PI3K inhibitor (LY294002) was obtained from MedChemExpress (USA).

### Total RNA isolation and gene cloning

Total RNA was extracted from HO8910 cells using RNAiso Plus (Takara, Dalian, China) and reverse-transcribed to cDNA using PrimeScriptRT reagent Kit with gDNA Eraser (Takara, Dalian, China) according to the manufacturer's instructions. The forward and reverse primers used for LAIR-1 mRNA transcript amplification were 5’-ATGTCTCCCCACCCCACCG-3’ and 5’-GGTCAGTGTCTGGCAACGGC-3'. The PCR product was purified from the gel using SanPrep Column DNA Gel Extraction Kit (Sangon Biotech, Shanghai, China), cloned into the T vector using pUCm-T Vector Rapid Cloning Kit (Sangon Biotech, Shanghai, China), and then transfected into E. coli DH5α cells which were grown in Luria broth (LB) medium supplemented with 100 μg/mL ampicillin. Recombinant clones were identified using colony PCR. The plasmids were purified from PCR positive colonies, using a TaKaRa MiniBEST Plasmid Purification Kit (Takara, Dalian, China), and sequenced by Sangon (Shanghai, China).

### Lentiviral production and establishment of stable expression cell lines

LAIR-1-lentivirus and LAIR-1-RNAi-lentivirus were constructed by GeneChem Company (Shanghai, China). To generate LAIR-1 construct, HO8910 cDNA was amplified by PCR and subcloned into the lentiviral vector pGC-Fu-3FLAG-CBh-gcGFP-IRES-EF1-Puromycin. The pFU-GW-016-shRNA-LAIR-1 lentiviral vector was generated according to our previous study [6]. The LAIR-1 shRNA targeting sequence was 5’-GGCCTTATCGCTGCATCTATT-3’, and the shRNA control sequence was 5’-TTCTCCGAACGTGTCACGT-3’. Forty-eight hours after transfection with constructed lentivirus, the cells were purified with 5μg/ml puromycin (Sigma-Aldrich) for one week. The observation of green fluorescence expression by fluorescence microscopy was used to assess infection effects. The LAIR-1 expression was confirmed by qRT-PCR and Western blotting.

### Western blotting analysis

Cells were lysed in RIPA lysis buffer (Beyotime, China) supplemented with protease inhibitors and phosphatase inhibitors (Roche, Mannheim, Germany) at 4°C for 30 min, and then centrifuged at 16,000×g for 15 min at 4°C to obtain the whole cell extract. Protein concentration was determined using a BCA protein assay kit (Beyotime, China). Cellular extracts were resolved by SDS-PAGE, transferred to PVDF membranes (Millipore, Billerica, USA), and blocked with 5% skim milk. The membranes were probed with the indicated primary antibodies at 4°C overnight, and then, incubated with the corresponding secondary antibodies at room temperature for 2 h. Corresponding antibody specific signals were detected by enhanced chemiluminescence kit (Pierce, USA) according to the manufacturer's instructions. GAPDH was used as a protein loading control.

### Quantitative real time PCR (qRT-PCR)

The Quantitative real-time PCR was used to evaluate the expression level of LAIR-1. The methods for obtaining total RNA and cDNA were described as above. Quantitative RT-PCR was performed using Thermo Scientific DyNAmo ColorFlash SYBR Green qPCR kit (F-416) according to the manufacturer's protocol. The forward and reverse primersused for lair-1 PCR amplification were 5'-TCTCCTCCTCCTGGTCCTCTTC-3' and 5'-GCCTTGTCTGCTGTCCT CTCTA-3'. House- keeping gene GAPDH was used as the internal control, the primers used for GAPDH were 5'- GTCTCCTCTGACTTCAACAGCG-3' and 5'-ACCACCCTGTTGCTGTAGCC AA-3'. The relative LAIR-1 mRNA expression levels were normalized against GAPDH using the comparative ΔΔCt method and relative fold change of gene was calculated by the equation 2 ^–ΔΔCt^. The primers for PCR were synthesized by Sangon (Shanghai, China).

### Cell proliferation, migration, apoptosis and colony formation assays

Cells were seeded in 96-well plates (1000 cells/well) and cell viability was examined using Cell Counting Kit-8 (CCK-8) assay (Dojindo Laboratories, Kumamoto, Japan). For colony formation assay, cells were seeded in 6-well plates (200 cells/well) and cultured under normal growth conditions for two weeks. Colonies were washed three times in pre-warmed PBS, fixed with 4% paraformaldehyde, stained with crystal violet, and counted using an inverted microscope. Migration assay was performed using the transwell system consisting of 24-well plate and 8-μm pore size with polycarbonate membrane (Corning Costar, Lowell, MA, U.S.A). Briefly, 5×10^4^ cells in FBS-free medium were plated in the top chamber, growth medium containing 10% FBS was used as a chemoattractant in the lower chamber. After 24 or 48 h, non-invaded cells on the upper surface of the filters were removed by wiping with a cotton swab and cells on the lower surface of the membranes were fixed with 4% paraformaldehyde and stained with crystal violet. Three different fields of the stained cells were photographed and counted under an inverted microscope at 100× magnification. For analysis of induction of apoptosis, cells were seeded into 6-well plates (1×10^5^/well) and cultured at 37°C, 5% CO_2_ for 24 h. Then cells were collected and stained with Annexin V-APC/PI (Keygen Biotechnology Co., Ltd., Nanjing, China) according to the product instructions. Subsequently, cells were immediately analyzed for apoptosis by flow cytometry (BD Biosciences, Franklin Lakes, NJ, USA). These experiments were with three replicates in each group and repeated three times.

### In vivo studies

Female athymic nude mice were purchased from Shanghai Laboratory Animal Center, CAS (Shanghai, China) and housed in a specific pathogen-free (SPF) environment. For in vivo tumorigenesis analysis, nude mice at the age of 5 weeks were randomly sorted into two groups (n = 6/each group), and were injected subcutaneously in the left flanks with 5 x 10^6^ of SKOV3 cells infected with LAIR-1-lentivirus (OE) or control-lentivirus (NC) in 0.1 mL serum-free PBS, respectively. Tumor growth was monitored at the indicated time points by measuring the length (L) and width (W) of the tumor, using a digital caliper, and tumor volume was calculated according to the following formula: volume = length × (width) ^2^/2. The mice were weighed every 2 days. After 14 days, the mice were sacrificed, and tumors were dissected and weighed. The percentages of growth inhibition were defined as the ratio of tumor weight to that in the vehicle control. All animal studies were performed with a protocol approved by the Institutional Animal Care and Use Committee of Binzhou Medical University.

### CO-IP assay

SKOV3 cells infected with LAIR-1-lentivirus (OE) or control-lentivirus (NC) were lysed in RIPA buffer supplemented with protease inhibitors and phosphatase inhibitors. The total cell extracts were centrifuged at 16,000 ×g for 15 min at 4°C, and the supernatant was incubated with anti-Flag antibody overnight at 4°C, then incubated with agarose beads (Santa Cruz, #sc-2003) at 4°C for 4 h, mouse IgG as a non-specific control. The resulting agarose beads were washed three times with 1×PBS and the Flag-associated and non-specific IgG-associated protein complexes were loaded into a 10% SDS-PAGE gel for electrophoresis. Proteins were stained with a coomassie brilliant blue staining solution and then destained with the destaining solution (Solarbio, #P1305).

### Sample preparation for MS analysis and LC-ESI-MS/MS Analysis by Q Exactive HF

The purified protein samples prepared from IP were separated by SDS-PAGE and subjected to LC-ESI-MS/MS analysis which was performed blindly by Micrometer Biotech Company (Hangzhou, China). In brief, the gel bands were cut into small pieces and destained. The proteins were reduced, alkylated, and digested by trypsin solution [25, 26]. Proteolysis was performed overnight at 37°C and stopped by adjusting the samples to 2% formic acid. The peptide in gel was extracted by 0.1% formic acid in acetonitrile.

The peptide was vaccum-dried in scanvac maxi-beta (Labogene), resuspended in 0.1% TFA and centrifuged at 20000 g for 2 min. The supernatant was transferred into sample tube and loaded onto an Acclaim PepMap 100 C18 trap column (Dionex, 75 μm×2 cm) by Ultimate 3000 nanoUPLC (Dionex) and the peptide was eluted onto an Acclaim PepMap RSLC C18 analytical column (Dionex, 75 μm×50 cm). A 70 min gradient was run at 280 nL/min, starting from 8 to 35% B (80% ACN, 0.1% FA), followed by 3 min linear gradient to 80% B, and maintenance at 80% B for 5 min. The peptides were subjected to NSI source followed by tandem mass spectrometry (MS/MS) in Q Oibitrap Fusion (Thermo Scientific) coupled online to the UPLC. Intact peptides were detected in the Orbitrap at a resolution of 60000. Peptides were selected for MS/MS using 25% NCE; ion fragments were detected in the Orbitrap at a resolution of 30000. A data-dependent procedure that alternated between one MS scan followed by 20 MS/MS scans was applied for the top20 precursor ions above a threshold ion count of 1E5 in the MS survey scan with 9 s dynamic exclusion. The electrospray voltage applied was 2.1 kV. Automatic gain control (AGC) was used to prevent overfilling of the ion trap; 3E6 and 5E4 ions were accumulated for generation of MS and MS/MS spectra separately. For MS scans, the m/z scan range was 350 to 1800 Da.

The resulting raw data was converted to mascot generic file with Proteome Discoverer (Thermo Scientific, v1.4.1.14) and processed using Mascot search engine (Matrix Science, v.2.3.02). Tandem mass spectra were searched against SwissProt human database (20350 sequences). Mass error was set to 10 ppm for precursor ions and 0.02 Da for fragment ions. Trypsin/P was selected for enzyme specificity and two missed cleavage was allowed. Carbamidomethylation on Cys was specified as fixed modification, oxidation on Met and acetylation on protein N-terminal were specified as variable modification. Decoy (reverse) database was searched against to estimate false discovery rate (FDR). The calculating results were revalued by algorithm percolator, and PSMs (Peptide-Spectrum Match) with p-value <0.05 and e-value <0.05 were accepted.

Gene Ontology (GO) enrichment analysis for LAIR-1 interacting proteins was conducted on a multi-omics data analysis website (http://www.omicsbean.cn). P value <0.05 was considered statistically significant.

### Statistical analysis

Data were expressed as mean ± SD. Student's t test was used to compare the values between subgroups. The statistical analyses were performed using the SPSS 16.0 software (SPSS). P -value <0.05 was considered significant.

## Supplementary Material

undefined
