# Supplementary material for: LAIR-1 suppresses cell growth of ovarian cancer cell via the PI3K-AKT-mTOR pathway
Source: Aging (Albany NY). 2020 Aug 31;12(16):16142–54. doi: 10.18632/aging.103589 (PMC7485720; doi:10.18632/aging.103589)
Supplement: undefined [file aging-12-103589-s001..pdf]

SUPPLEMENTARY FIGURE

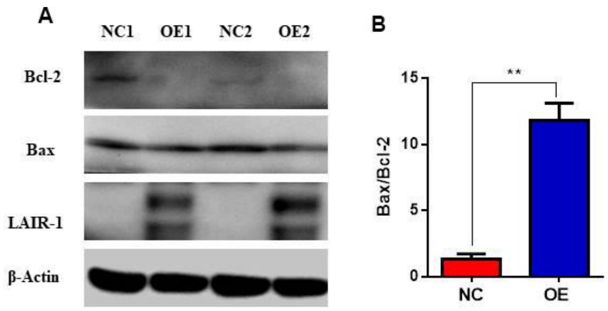

Supplementary Figure 1. LAIR-1 promotes ovarian carcinoma cell apoptosis in vivo. (**A**) Western blot analyses of Bcl-2, Bax, LAIR-1 in tumor tissues derived from control and LAIR-1 overexpression SKOV3 cells. β- Actin was used as a loading control.
